# Therapeutic efficacy of dienogest combined with GnRH agonist in adenomyosis and associated obstetric risk factors: A meta-analysis

**DOI:** 10.1097/MD.0000000000045907

**Published:** 2025-11-28

**Authors:** Yan Wang, Xiye Wang, Leyi Zhang

**Affiliations:** a Department of Gynaecology, Shengzhou People’s Hospital, Shaoxing, Zhejiang Province, China.

**Keywords:** adenomyosis, dienogest, GnRH-a, MA, risk factors

## Abstract

Adenomyosis is a chronic gynecological condition commonly affecting women of reproductive age, with its etiology still unclear. In clinical practice, gonadotropin-releasing hormone agonists (GnRH-a), often combined with other drugs, are used to treat mild to moderate cases. This meta-analysis aimed to evaluate the therapeutic efficacy of dienogest combined with GnRH-a in managing adenomyosis and explore related obstetric risk factors. A systematic search was conducted for relevant studies published up to 2024, with 5 studies involving 520 patients included in the meta-analysis. Results showed that compared to monotherapy, the combination of dienogest and GnRH-a significantly improved visual analogue scale scores, hemoglobin levels, CA-125 levels, and uterine volume, with no significant difference in adverse event rates. Additionally, 11 studies with 15,015 participants analyzed obstetric risk factors, revealing that women with adenomyosis had significantly higher risks of spontaneous abortion, premature rupture of membranes, preterm birth, small-for-gestational-age fetuses, and cesarean section. These findings suggest that dienogest combined with GnRH-a can improve therapeutic outcomes for adenomyosis, while also highlighting increased obstetric risks associated with the condition.

## 1. Introduction

Adenomyosis is a benign uterine disease with growth function of endometrial stroma and glands, which grows and invades the myometrium under the stimulation of multiple pathogenic factors.^[1]^ Adenomyosis has a high incidence in women of childbearing age, and most of them show the characteristics of increased menstrual volume, prolonged menstrual period, dysmenorrhea, and infertility.^[2]^ Hysterectomy is an effective method to treat the disease, but for patients with fertility preservation, it is important to find a more effective and suitable treatment.

In clinical practice, gestrinone is often used for the treatment of adenomyosis. This drug can improve dysmenorrhea by suppressing the effect of estrogen and affecting the survival state of cells in the lesion.^[3]^ However, gestrinone has many side effects, and the curative effect is not ideal. Dienogest is a new contraceptive drug developed by German Jenapharm company. It is a progestogen drug, which has a strong effect of suppressing ovulation. Dienogest has strong progesterone activity, which only binds to progesterone receptor, can reduce endogenous estrogen production, and inhibit the stimulation of estrogen on normal or ectopic endometrium.^[4]^ Dienogest has mainly peripheral effects, similar to naturally produced progesterone, which is beneficial to the endometrium.^[5]^ Guo et al ^[6]^ revealed that dienogest used in the clinical treatment of people with adenomyosis can visibly improve the symptoms of dysmenorrhea, and effectively control the growth of uterine volume (UV) and endometrial thickening. However, dienogest has certain side effects, including irregular vaginal bleeding, amenorrhea, prolonged menstruation, and emotional changes. Gonadotropin-releasing hormone agonist (GnRH-a) is a synthetic decapeptide compound, which can efficiently bind to GnRH receptor and inhibit ovarian secretion of ovarian estrogen and luteinizing hormone through negative feedback, and then maintain a sustained and low concentration of estrogen in the body.^[7,8]^ GnRH-a can long-term eliminate the stimulatory effect of estrogen on adenomyosis, and hinder or narrow the lesions.^[9]^ GnRH-a can also improve the receptivity of endometrium to embryos by suppressing the synthesis and release of various cells or immune factors, which is conducive to the development and maturation of oocytes and plays a role in preventing the recurrence of adenomyosis.^[10]^ GnRH-a long-term medication will also produce side effects, including perimenopausal symptoms and osteoporosis caused by low estrogen levels, as well as ovarian dysfunction. In summary, both dienogest and GnRH-a can be used in adenomyosis, and the curative effect is obvious, but both of them have certain side effects.

To understand the effect of dienogest plus GnRH-a in adenomyosis, this article included the relevant literatures and systematically evaluated the efficacy and safety of dienogest plus GnRH-a in adenomyosis through MA, and analyzed the risk factors of adverse pregnancy outcomes of adenomyosis. It provides the basis for the choice of clinical treatment of adenomyosis and improve the adverse pregnancy outcomes of patients.

## 2. Materials and methods

### 2.1. Selection criteria

This research was approved by the Ethics Committee of Shengzhou People’s Hospital.

Inclusion: clinical controlled studies, ignoring whether allocation was concealed or blinded; Subjects with clinical diagnosis and confirmed adenomyosis, regardless of race; The intervention measures were dienogest or GnRH-a single drug and dienogest combined with GnRH-a; and the VAS score of dysmenorrhea, hemoglobin (Hb), CA-125, UV, and incidence of AE as outcome indicators in the study of efficacy analysis; adverse pregnancy was the outcome index in the risk factor analysis.

Exclusion: duplicate publications; literature review or MA; the sample size is too small; basic experiments; case or experience report; the original data cannot be extracted; and articles unable to get full text.

### 2.2. Search strategy

The approach of integrating subject terms with free words was employed to retrieve pertinent literature. First, the relevant literature was retrieved from PubMed, EBbase, etc with the search terms of “Dienogest,” “Gonadotropin-releasing hormone antigonis,” “GnRH-a,” “Adenomyosis,” “Endometriosis,” “Endometriose,” and “Endometriomas,” duration: the establishment of the database to May 2024, and the language was unlimited. Subsequently, Google Scholar, SCI-HUB, and other search engines were adopted for searching.

### 2.3. Screening, data extraction, and evaluation

Two researchers independently completed the literature screening, data extraction, and evaluation, and performed cross-check. If there was any disagreement, it would be decided by discussing or inviting the third researcher to participate in the discussion.

The titles and abstracts of the retrieved articles were read, and the articles that obviously did not meet the requirements were excluded. Secondly, the full text of the article that initially met the requirements was downloaded and read, and the article that was included in this MA was finally determined. The basic data (title, author), research data (subjects, sample size), efficacy analysis outcome indicators (dysmenorrhea VAS score, Hb, CA-125, UV, and incidence of AE), and adverse pregnancy outcome indicators (abortion, premature rupture of membranes [PRM], preterm birth [PTB], small for gestational age [SGA] fetuses, and cesarean section [CS]) of the included article were extracted. The tool recommended by the Cochrane Collaboration was used for methodological evaluation, including random sequence generation, allocation consensus, blinding of participants and personnel, blinding of outcome assessment, incomplete outcome data, selective reporting, and others. “High,” “low,” and “unclear” risk were judged.

### 2.4. Statistical processing

RevMan 5.3 software was utilized to conduct MA. For the outcome analysis, the measurement data were reported using mean difference (MD) and 95% confidence interval (CI), and the count data were reported using odds ratio (OR) and 95% CI. In the analysis of risk factors, the random effects inverse variance weighting model was used to summarize OR, and the log (OR) and standard error (SE) were calculated by calculator function. After merging each log (OR) and SE, the combined OR with 95% CI was calculated. Subgroup analysis and *I*^2^ test were adopted for analyzing the heterogeneity (Het) cross the studies. When there was obvious Het (*I*^2^ > 50% and *P < *.10), the source of Het needed to be clarified. If there was only statistical Het but no clinical Het, the random effect model (REM) was utilized. When there was no obvious Het (*I*^2^ ≤ 50% and *P* ≥ .10), the fixed effect model (FEM) was utilized. Funnel plot was drawn for publication bias (PB) analysis. The test level of MA was α = 0.05.

## 3. Results

### 3.1. Literatures screening

**Figure 1.**
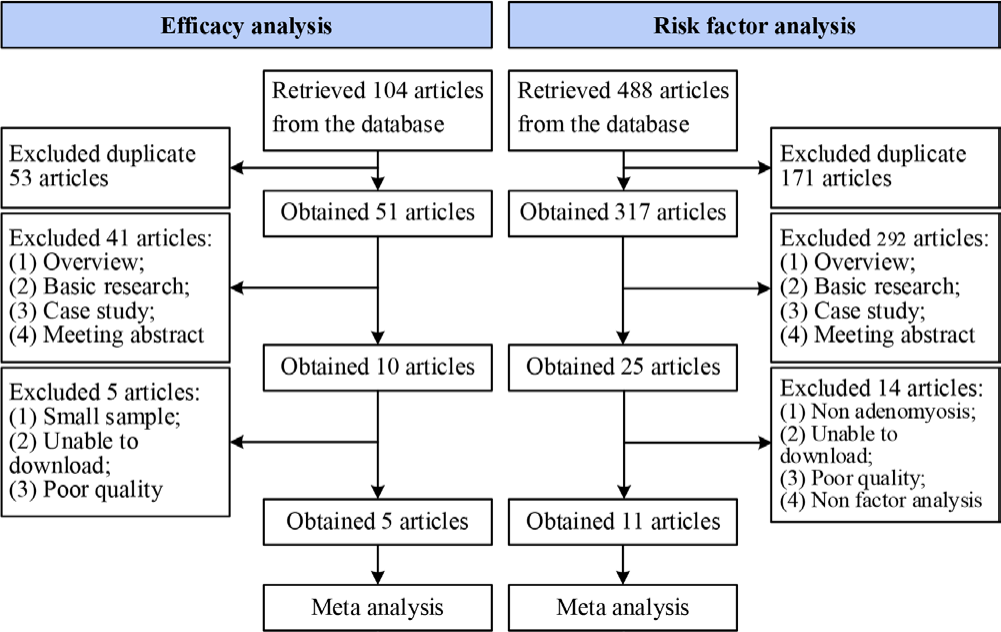
Literatures selection process.

One hundred and four articles about dienogest plus GnRH-a in adenomyosis were retrieved from the databases, and 53 repeatedly published articles were excluded, and 51 articles were included for preliminary screening; 41 reviews, basic research, case studies, and conference abstracts were excluded, and 10 were included for in-depth screening; Five articles with small samples, unable to download, with poor quality were excluded, and 5 articles^[11–15]^ were finally included in the MA. 488 articles related to the analysis of risk factors of adenomyosis were obtained, 171 duplicate articles were excluded, and 317 articles were included for preliminary screening; 292 reviews, basic research, case studies, and conference abstracts were excluded, and 25 were included for in-depth screening; Excluding 14 articles, 11 articles^[16–26]^ were finally included in the MA for outcome contrast (Fig. 1).

### 3.2. Fundamental features

Table 1 presents the fundamental features of the 5 included articles, including 520 subjects, 234 subjects received dienogest or GnRH-a monotherapy, and 286 subjects received dienogest and GnRH-a; The outcome measures were VAS, Hb, CA-125, UV, and AE rate. The fundamental features of the 11 studies are presented in Table 2, including 15,015 subjects, 1481 subjects with adenomyosis and 13,534 subjects without adenomyosis; The study designs were of Cohort type; The risk factors were delivery, abortion, PRM, PTB, SGA fetuses, and CS.

**Table 1:** Fundamental features of articles related to the remedy of adenomyosis with dienogest plus GnRH-a.

Author	Year	Intervention mode		Sample size		Outcome measures
		Two-drug	Single-drug	Two-drug	Single-drug	
Chan^[11]^	2023	After 6 mo of GnRH-a remedy, dienogest 2 mg/d	11.25 mg GnRH-a, 6 months	44	46	VAS; Hb; CA-125; UV; AE
Matsushima^[12]^	2020	After 6 mo of GnRH-a remedy, dienogest 2 mg/d	1.88 mg GnRH-a, 4 wk/time, 6 mo	15	15	Hb; CA-125; UV; AE
Miao^[13]^	2022	After 4 mo of remedy with 3.75 mg GnRH-a (4 wk/time), dienogest 2 mg/d	Dienogest 2 mg/d	71	52	CA-125; UV; AE
Wang^[14]^	2023	After GnRH-a remedy, 1 tablet/d of dienogest	3.6 mg GnRH-a for 6 cycles	60	60	VAS; CA-125
Zhu^[15]^	2023	Dienogest 2 mg/d, 3–6 doses of GnRH-a	Dienogest 2 mg/d	96	61	Hb; CA-125; UV; AE

AE = adverse event, CA-125 = cancer antigen 125, CS = cesarean section, Hb = hemoglobin, UV = uterine volume, VAS = visual analogue scale.

**Table 2:** Fundamental features of articles related to risk factors of adenomyosis.

Author	Year	Design	Study subjects		Sample size		Risk factors
			Observation group	Control group	Observation group	Control group	
Exacoustos^[16]^	2016	Cohort	Adenomyosis	Normal	200	300	Abortion; Preterm delivery; SGA fetuses; CS
Genc^[17]^	2015	Cohort	Adenomyosis	Non adenomyosis	327	618	Delivery; abortion
Güzel^[18]^	2015	Cohort	Adenomyosis	Normal	26	22	Delivery; abortion
Hashimoto^[19]^	2018	Cohort	Adenomyosis	Non adenomyosis	49	245	Abortion; Preterm delivery; SGA fetuses
Joachim^[20]^	2023	Cohort	Adenomyosis	Non adenomyosis	386	323	Delivery
Juang^[21]^	2007	Cohort	Adenomyosis	Non adenomyosis	35	277	PRM; PTB
Mochimaru^[22]^	2015	Cohort	Adenomyosis	Non adenomyosis	36	144	Delivery; abortion; PRM; Preterm delivery; SGA fetuses; CS
Romanek^[23]^	2010	Cohort	Combined with adenomyosis	Uterine leiomyoma	135	176	Delivery; abortion; CS
Shin^[24]^	2018	Cohort	Adenomyosis	Non adenomyosis	47	8057	Abortion; Preterm delivery; CS
Shinohara^[25]^	2020	Cohort	Adenomyosis	Non adenomyosis	61	244	PRM; Preterm delivery; SGA fetuses; CS
Trinchant^[26]^	2022	Cohort	Adenomyosis	Non adenomyosis	179	3128	Delivery; abortion; Preterm delivery; CS

CS = cesarean section, PRM = premature rupture of membranes, PTB = preterm birth, SGA = small for gestational age.

### 3.3. Assessment

**Figure 2.**
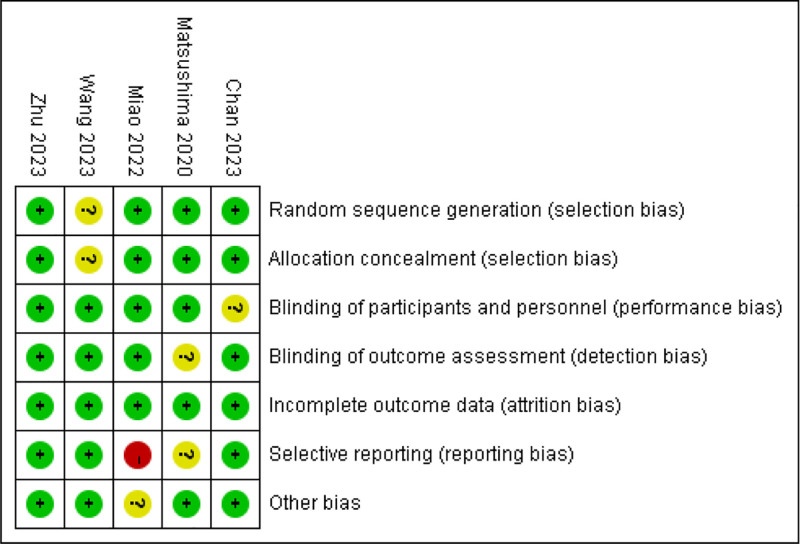
Risk of bias assessment.

Among the 5 articles, 1 selectively reported the outcome indicators, so it was evaluated as “high risk.” The entries of other studies were evaluated as “low” or “unclear risk,” respectively (Fig. 2).

### 3.4. MA results of dienogest plus GnRH-a in adenomyosis

#### 3.4.1. VAS score

**Figure 3.**
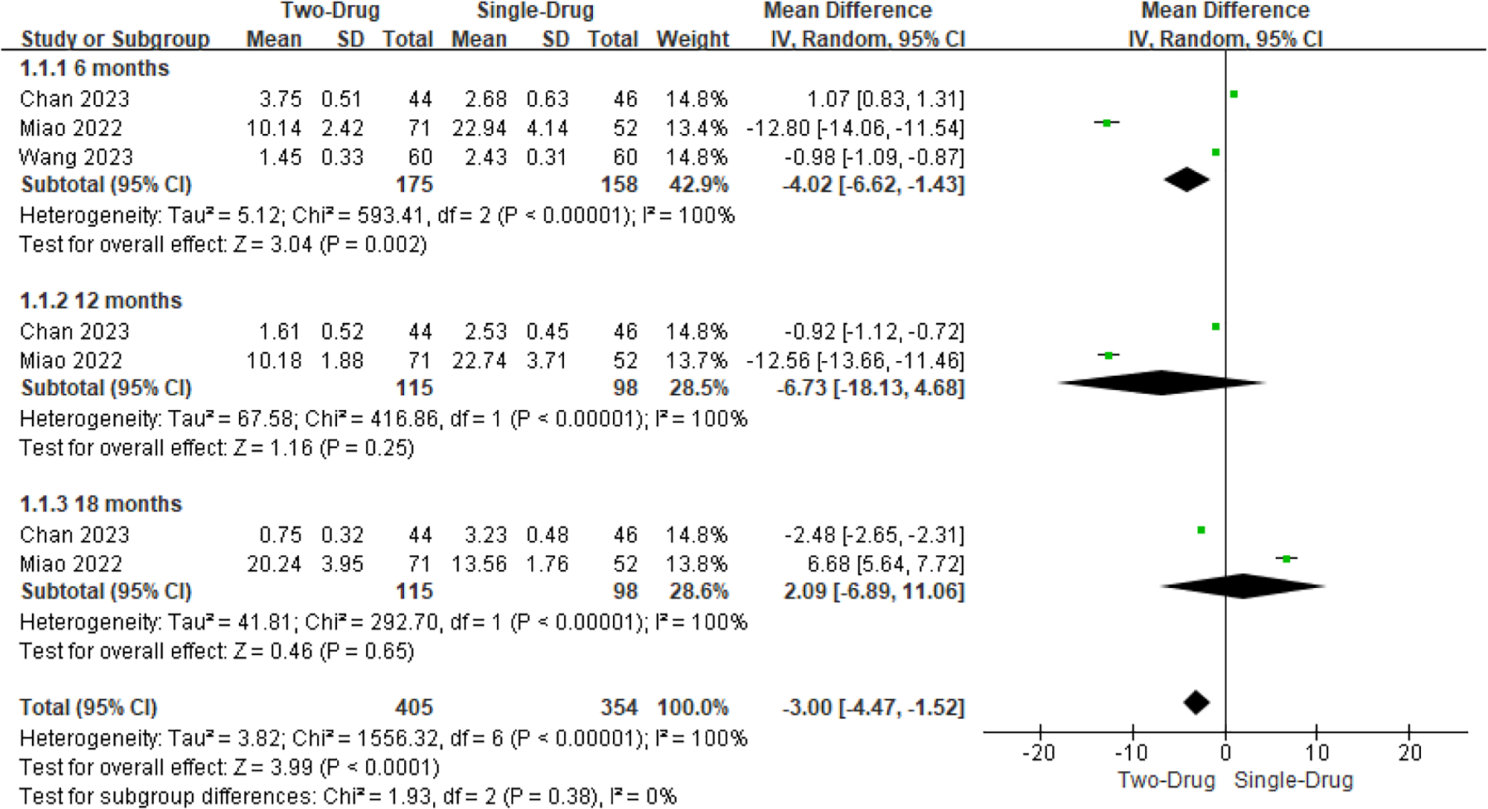
MA forest plot (FOP) of VAS score contrast after medication. MA = meta-analysis, VAS = visual analogue scale

Two to 3 studies involved VAS scores after single drug and combination of 2 drugs. Het was evident across the studies (*I*^2^ = 100%, *P < *.00001). Consequently, a REM was utilized for analysis. The results of subgroup analysis revealed that the VAS score of the combination of the 2 drugs at 6 months was visibly lower as against the single drug (MD = −4.02, 95% CI: −6.62 to −1.43, *P = *.002), while there was similar between the VAS scores at 12 and 18 months (*P > *.05). On the whole, the VAS score after the combination of the 2 drugs was visibly lower as against the single drug (−3.00, −4.47 to −1.52, <.0001) (Fig. 3).

#### 3.4.2. Hb

**Figure 4.**
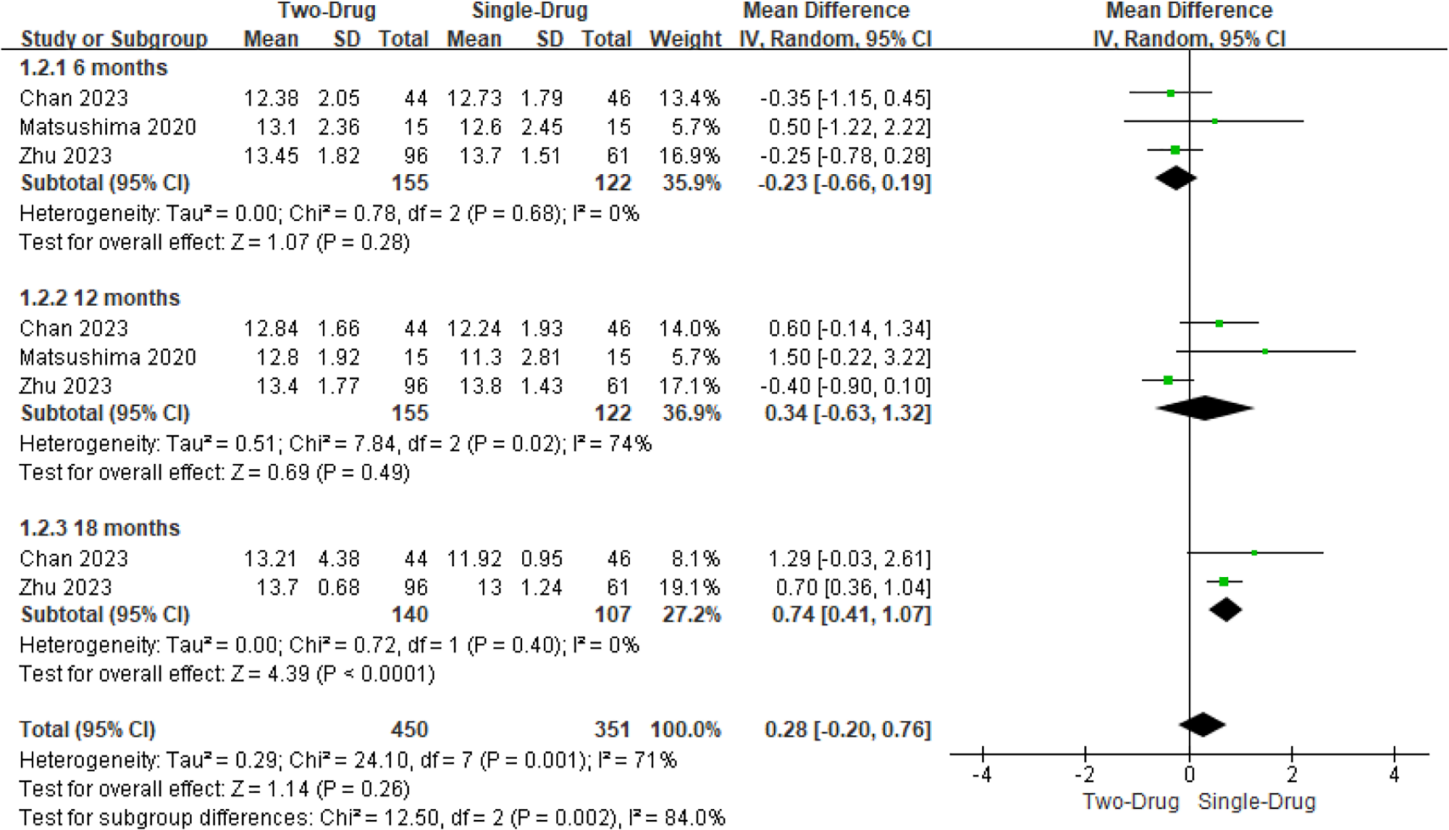
FOP of MA for contrast of Hb after medication. MA = meta-analysis.

Two to 3 studies involved the Hb after single drug and combination of 2 drugs. Visible Het was observed (*I*^2^ = 71%, *P = *.001). Consequently, a REM was utilized. It revealed that the Hb 18 months after the combination of the 2 drugs was visibly higher as against the single drug (MD = 0.74, 95% CI: 0.41 to 1.07, *P < *.0001), while there was similar in the Hb 6 months and 12 months after remedy (*P > *.05). Hb had no obvious distinction following remedy on the whole (0.28, −0.20 to 0.76, .26) (Fig. 4).

#### 3.4.3. CA-125

**Figure 5.**
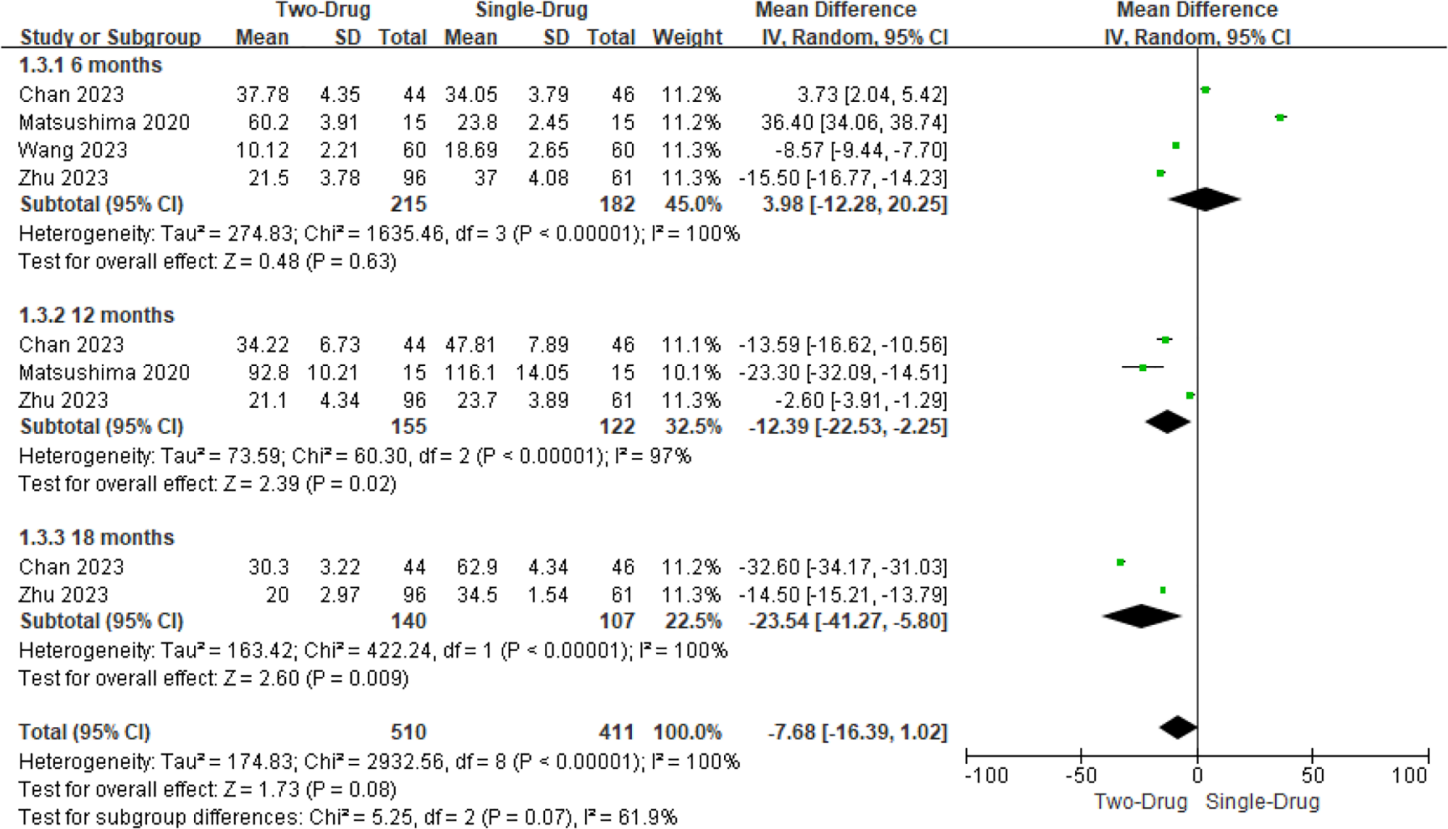
MA FOP of contrast of CA-125 following administration. CA-125 = cancer antigen 125, MA = meta-analysis.

Two to 4 studies involved the level of CA-125 after administration. Visible Het was observed (*I*^2^ = 100%, *P < *.00001). Consequently, a REM was utilized. It revealed that the level of CA-125 at 12 and 18 months after the combination of the 2 drugs was visibly lower as against the single drug (MD = −12.39, 95% CI: −22.53 to −2.25, *P = *.002; −23.54, −41.27 to −5.80, .009), while there was similar in the level of CA-125 at 6 months (*P > *.05). On the whole, the level of CA-125 had no visible distinction following remedy (−7.68, −16.39 to 1.02, 0.08) (Fig. 5).

#### 3.4.4. UV

**Figure 6.**
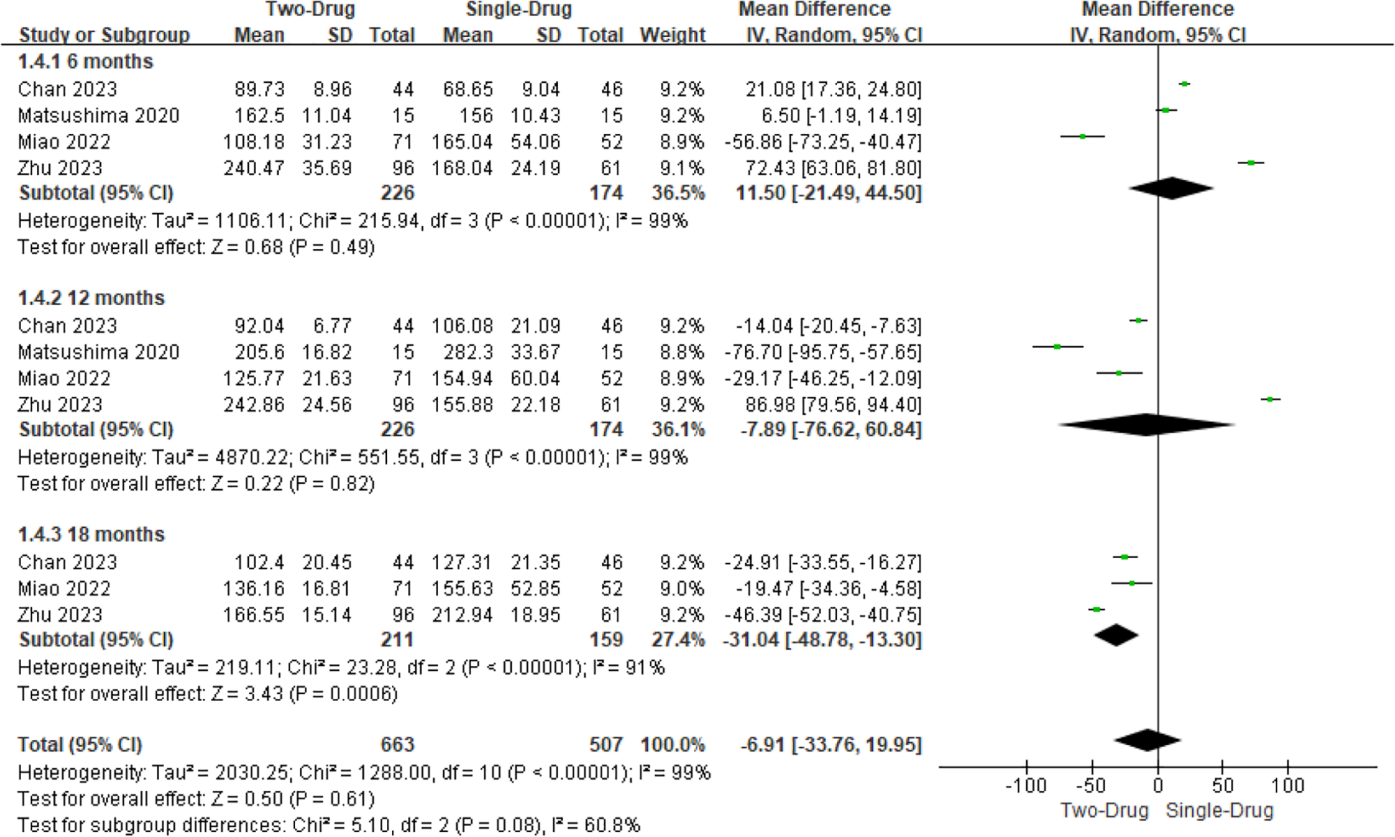
FOP of MA for contrast of UV following administration. MA = meta-analysis.

Three to 4 studies involved the UV following administration. Visible Het was observed (*I*^2^ = 99%, *P < *.00001). Consequently, a REM was utilized. It revealed that the UV at 18 months following the combination of the 2 drugs was visibly smaller as against the single drug (MD = −31.04, 95% CI: −48.78 to −13.30, *P = *.0006), while there was similar in the UV at 6 months and 12 months (*P > *.05). UV had no obvious distinction following remedy (−6.91, −33.76 to 19.95, .61) (Fig. 6).

#### 3.4.5. AE rate

**Figure 7.**
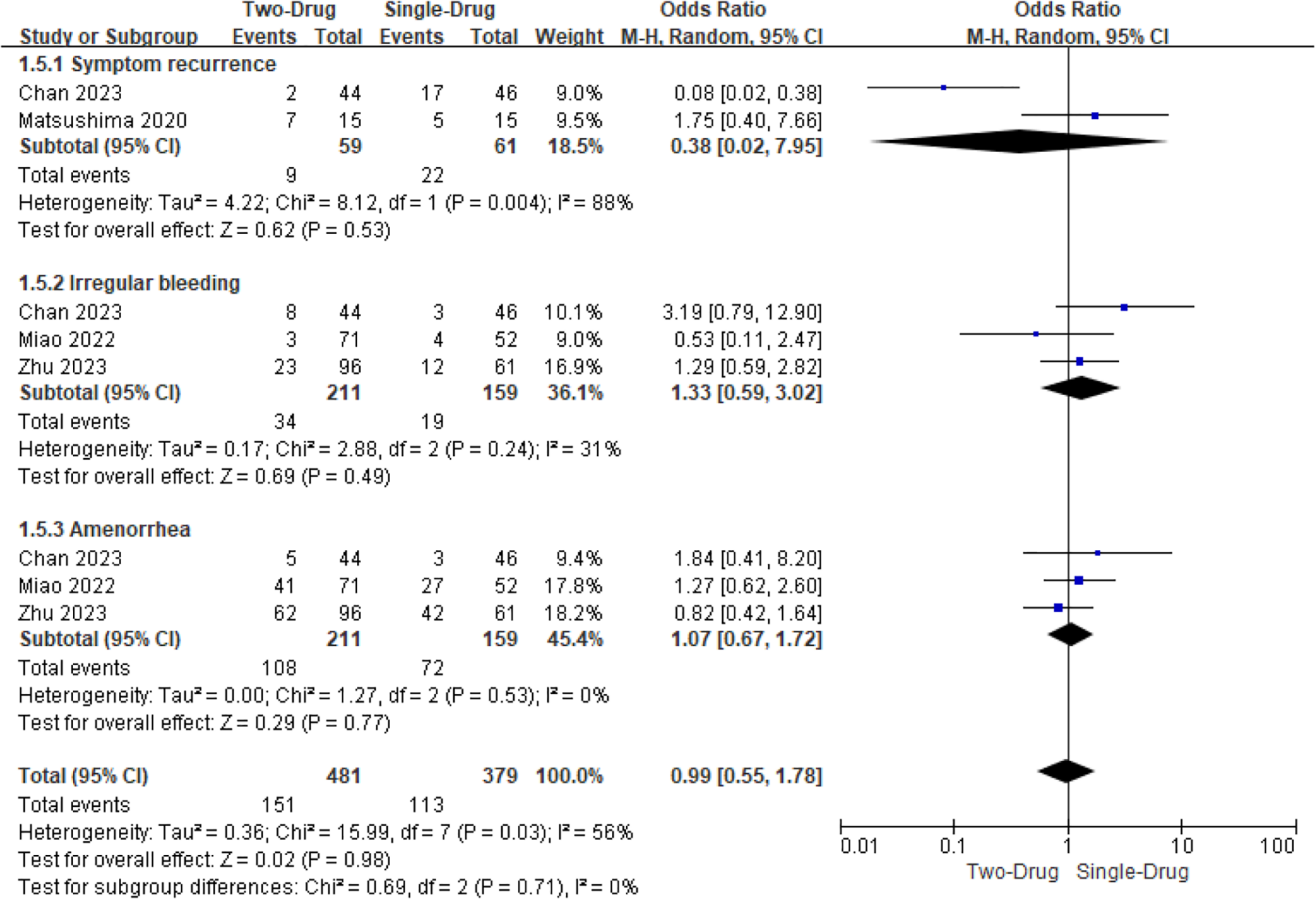
MA FOP for contrast of AE rates following medication. AE = adverse event, MA = meta-analysis.

Three studies involved the occurrence of AE following administration. Visible Het was observed (*I*^2^ = 56%, *P = *.03). Consequently, a REM was utilized. The subgroup and overall analysis revealed that there was similar in the occurrence of AE following administration (OR = 0.99, 95% CI: 0.55, 1.78, *P = *.98) (Fig. 7). The most frequently reported AEs included irregular vaginal bleeding, amenorrhea, hot flashes, and mood changes, consistent with the known safety profiles of dienogest and GnRH-a. No severe or unexpected AEs were reported.

### 3.5. MA of risk factors related to adenomyosis

#### 3.5.1. Delivery history

**Figure 8.**
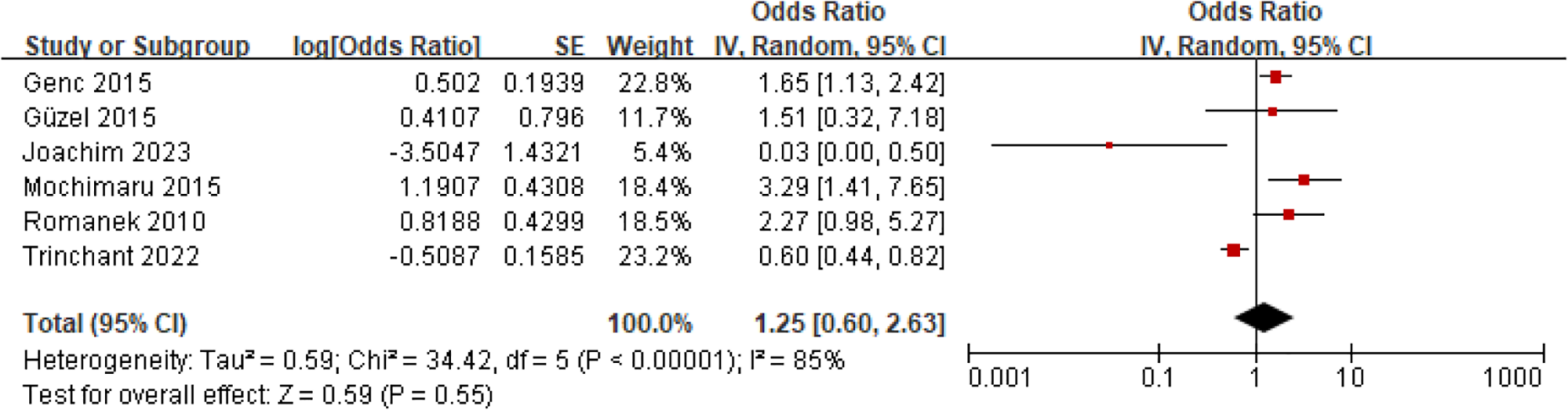
FOP of MA on the relationship between adenomyosis and normal delivery. MA = meta-analysis.

Six studies involved the relationship between normal delivery and adenomyosis. Visible Het was observed (*I*^2^ = 85%, *P < *.00001). Consequently, an REM was utilized. MA revealed that there was similar in the normal delivery rate between people with adenomyosis and people without adenomyosis (OR = 1.25, 95% CI: 0.60–2.63, *P = *.55) (Fig. 8).

#### 3.5.2. Abortion history

**Figure 9.**
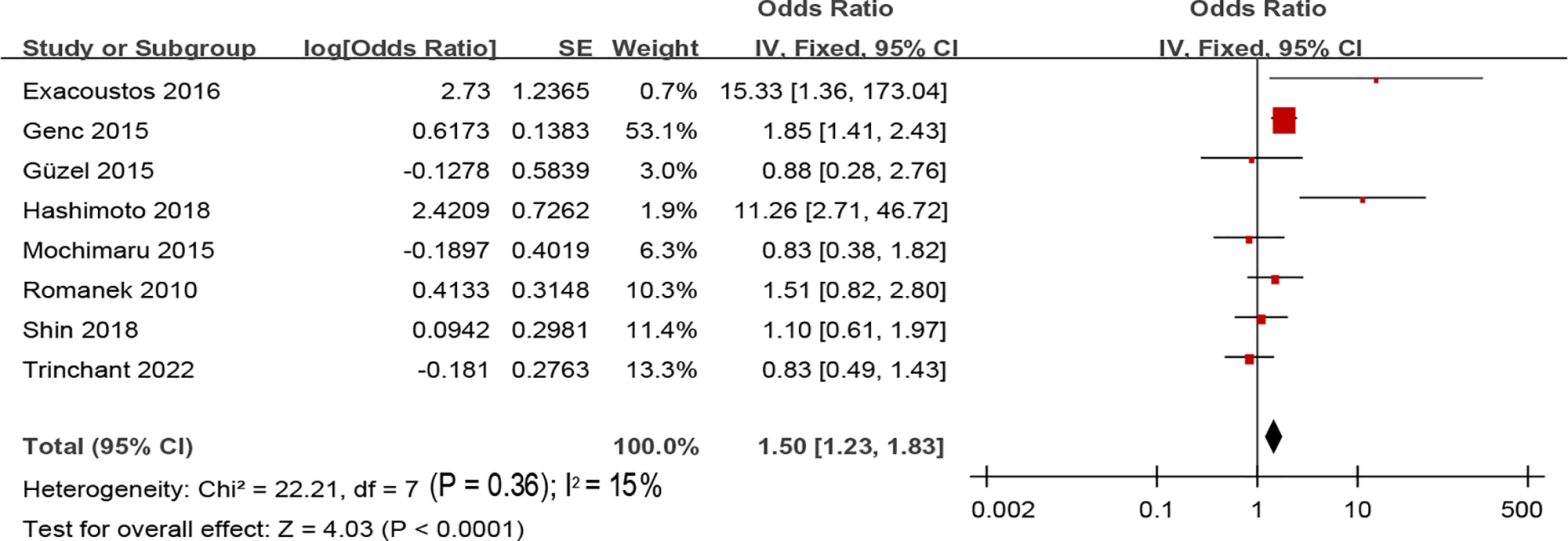
FOP of MA on the relationship between adenomyosis and abortion.

Eight studies involved the relationship between abortion and adenomyosis, without obvious Het cross studies (*I*^2^ = 15%, *P = *.36). Consequently, a FEM was utilized. MA suggested that the abortion rate of people with adenomyosis was visibly higher as against people without adenomyosis (OR = 1.50, 95% CI: 1.23–1.83, *P < *.0001) (Fig. 9).

#### 3.5.3. History of PRM

**Figure 10.**
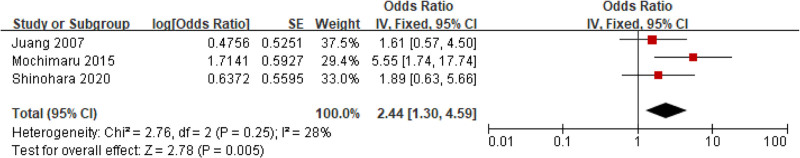
FOP of MA on the relationship between adenomyosis and PRM. MA = meta-analysis, PRM = premature rupture of membranes.

Three studies involved the relationship between PRM and adenomyosis, without clear Het cross studies (*I*^2^ = 28%, *P = *.25). Consequently, a FEM was utilized. MA suggested that the rate of PRM in people with adenomyosis was markedly higher as against people without adenomyosis (OR = 2.44, 95% CI: 1.30–4.59, *P = *.005) (Fig. 10).

#### 3.5.4. History of PTB

**Figure 11.**
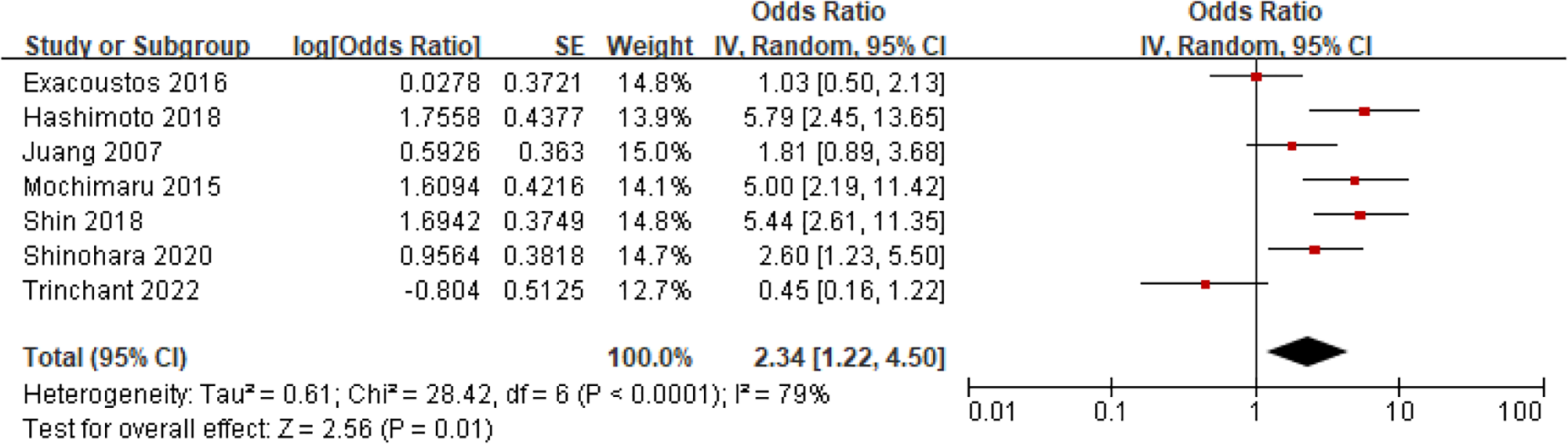
FOP of MA on the relationship between adenomyosis and PTB. MA = meta-analysis, PTB = preterm birth.

Seven studies involved the relationship between PTB and adenomyosis, and Visible Het was observed (*I*^2^ = 79%, *P < *.0001). Consequently, a REM was utilized. MA suggested that the previous PTB rate of people with adenomyosis was markedly higher as against people without adenomyosis (OR = 2.34, 95% CI: 1.22–4.50, *P = *.01) (Fig. 11).

#### 3.5.5. History of SGA fetuses

**Figure 12.**
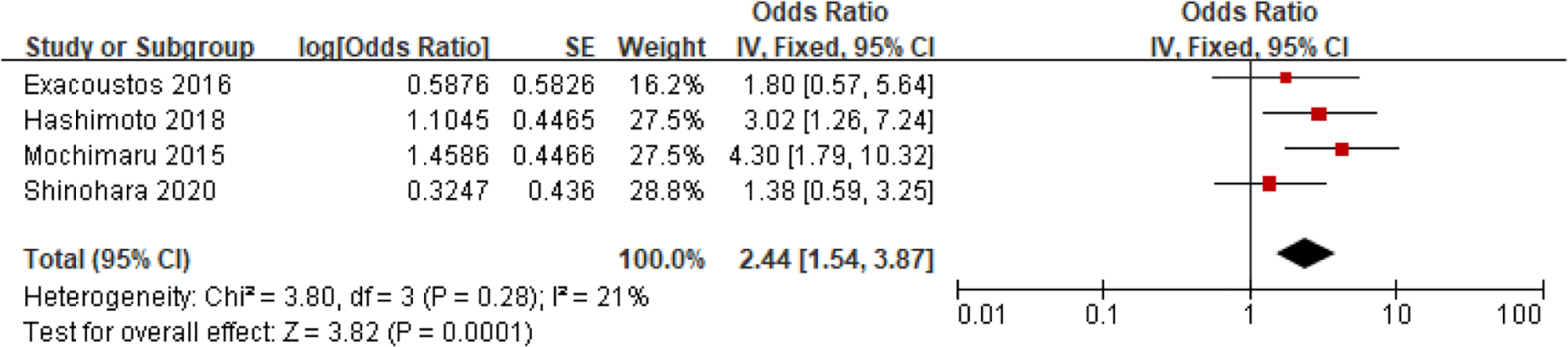
FOP of MA of the relationship between adenomyosis and SGA fetuses. MA = meta-analysis, SGA = small for gestational age.

Four studies involved the relationship between SGA fetuses and adenomyosis, without clear Het cross studies (*I*^2^ = 21%, *P = *.28). Consequently, a FEM was utilized. MA suggested that the rate of SGA fetuses in people with adenomyosis was markedly higher as against people without adenomyosis (OR = 2.44, 95% CI: 1.54–3.87, *P = *.0001) (Fig. 12).

#### 3.5.6. History of CS

**Figure 13.**
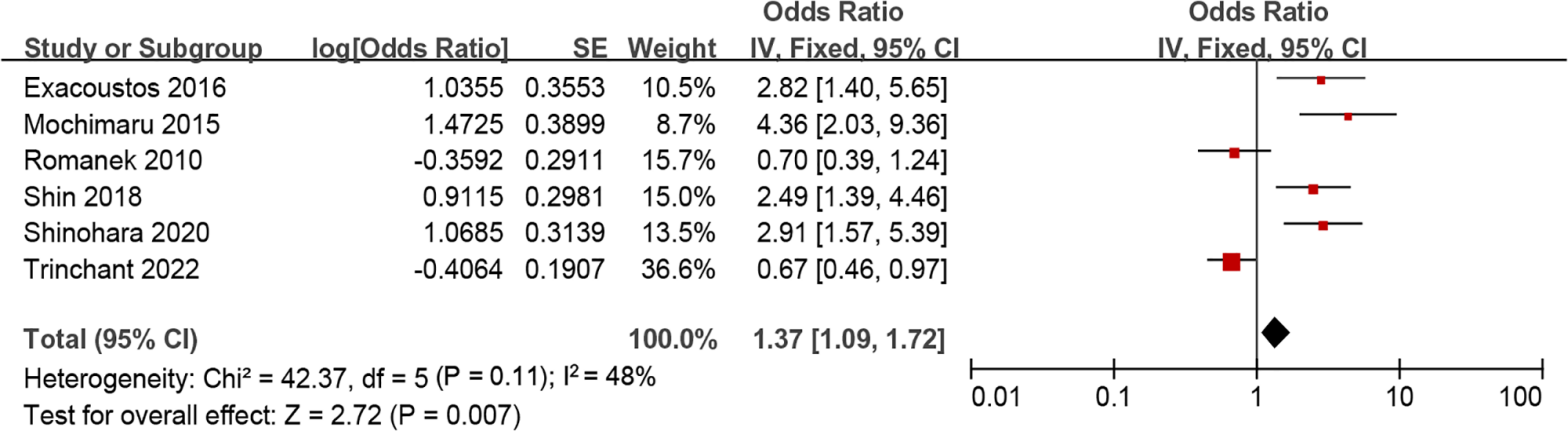
FOP of MA on the relationship between adenomyosis and CS. CS = cesarean section, MA = meta-analysis.

Six studies involved the relationship between CS and adenomyosis, without clear Het cross studies (*I*^2^ = 48%, *P = *.11). Consequently, a FEM was utilized. MA suggested that the CS rate of people with adenomyosis was markedly higher as against people without adenomyosis (OR = 1.37, 95% CI: 1.09–1.72, *P = *.007) (Fig. 13).

### 3.6. PB

**Figure 14.**
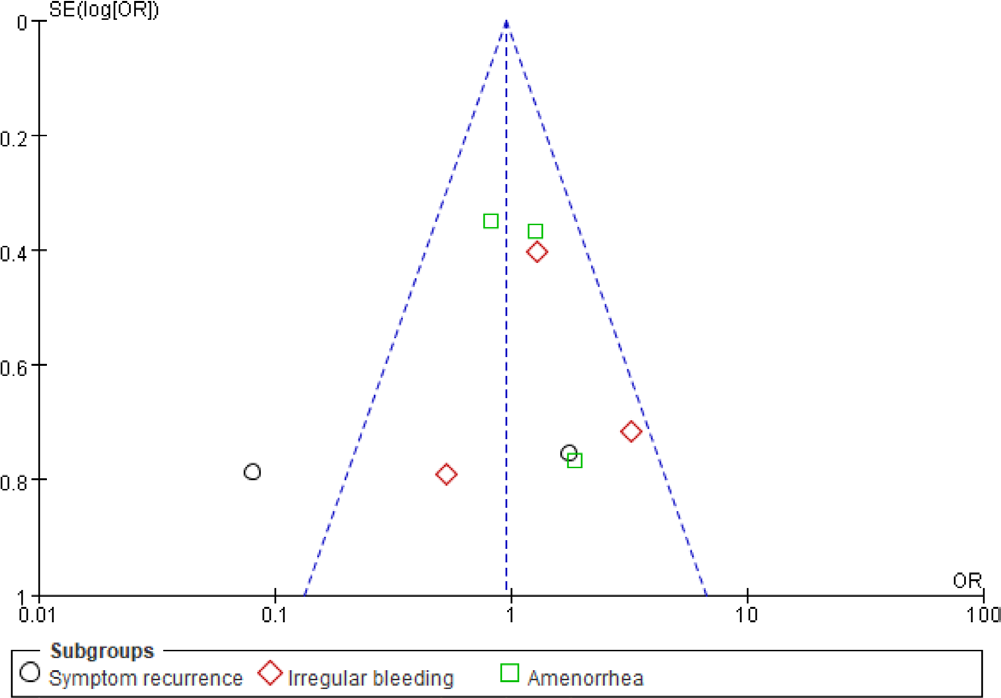
MA FUP of AE. AE = adverse event, FUP = funnel plot, MA = meta-analysis.

**Figure 15.**
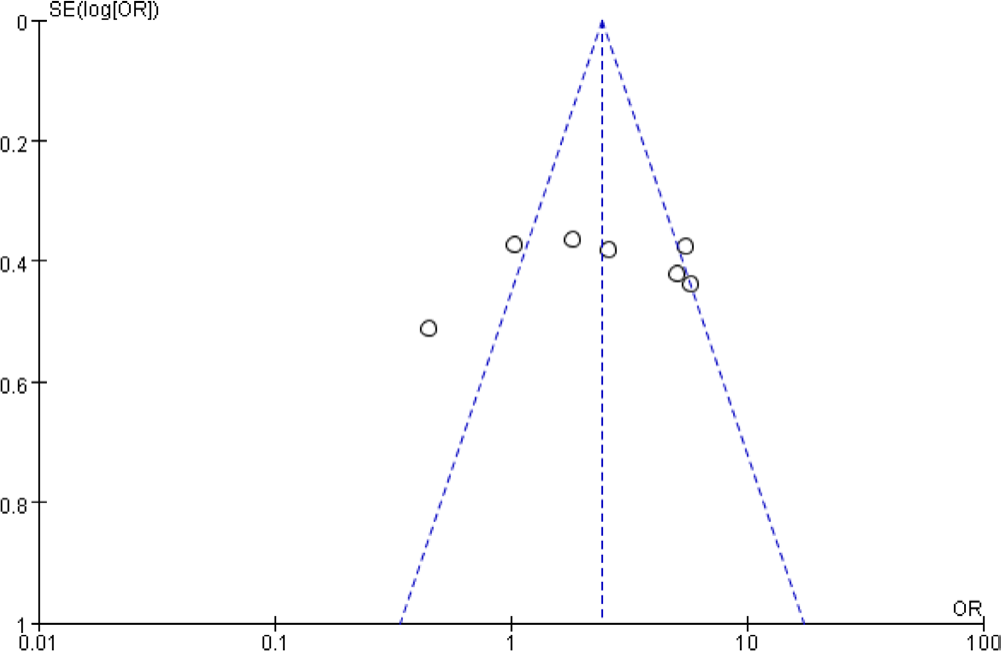
MA FUP of PTB. FUP = funnel plot, MA = meta-analysis, PTB = preterm birth.

Taking the outcome indicators of AE following dienogest plus GnRH-a in adenomyosis and the analysis indicators of the relationship between PTB and adenomyosis as an example, the funnel plot of MA was drawn to analyze the PB. The SE of the included studies was relatively small, and the studies were evenly distributed on both sides of the vertical line, and few studies fell outside the 95% CI area (outside the slash). Therefore, it was believed that the included studies had small PB (Figs. 14 and 15).

## 4. Discussion

Adenomyosis is a benign uterine disease due to the growth of endometrial glands and stroma and invasion of the myometrium, accompanied by smooth muscle hyperplasia.^[27,28]^ At present, the etiology of adenomyosis is not clear, and its incidence gradually shows a trend of youth, so it is very important to find a reasonable and effective treatment. However, dienogest and GnRH-a can be utilized in the clinical treatment of adenomyosis, but the curative effect changes following the combination of the 2 drugs still need a lot of clinical evidence to verify. To fill this gap, this MA first included the relevant literature, and systematically evaluated the efficacy and safety of dienogest plus GnRH-a in adenomyosis.

Dysmenorrhea is the main clinical feature of people with adenomyosis, and most of them show progressive menstrual pain, increased menstrual volume, and prolonged menstrual period.^[29]^ This MA found that as against monotherapy, the dysmenorrhea pain score of people with adenomyosis following dienogest and GnRH-a was markedly reduced. The results suggested that dienogest plus GnRH-a in adenomyosis can effectively relieve the symptoms of dysmenorrhea in patients. This is because dienogest has similar pharmacological effects to endogenous progestogens, which can specifically integrate progesterone derivatives and ethylene nortestosterone to promote endometrial tissue stability, and play a role in relieving pain and improving clinical symptoms.^[30]^ GnRH-a can inhibit the synthesis and release of various cytokines and immune factors in peritoneal fluid, improve the intraperitoneal environment, and improve clinical symptoms such as dysmenorrhea.^[31]^ Secondly, this MA found that as against the single drug treatment, the Hb of people with adenomyosis was markedly increased and the level of CA-125 was markedly decreased following dienogest and GnRH-a for 18 months. When the condition of adenomyosis is serious, the patient’s endometrium is dysfunctional, causing an increase in menstrual volume, which leads to anemia and shows a decrease in Hb. CA-125 is a mucin like glycoprotein, which is mainly distributed in mesothelial cells and tissues. However, CA-125 in peripheral blood of people with adenomyosis is abnormally elevated, and CA-125 can be adopted to assess the UV and the degree of residual lesions following surgery.^[32,33]^ Dienogest plus GnRH-a can hinder or shrink the lesion tissue by reducing the level of CA-125 in adenomyosis, which is very important to suppress the recurrence following treatment. Adenomyosis is due to the invasion of endometrial glands and stroma into the myometrium, which will cause the increase of UV and uterine cavity area to a certain extent, and also cause uterine contractile dysfunction.^[34,35]^ Ali et al ^[36]^ found that dienogest utilized in adenomyosis can markedly improve the symptoms of dysmenorrhea and chronic pelvic pain, but the therapeutic outcome on UV is not obvious. However, this MA found that as against monotherapy, the UV of adenomyosis patients treated with dienogest and GnRH-a for 18 months was markedly reduced. GnRH-a can act on the hypothalamic pituitary gonadal axis and reduce the UV.^[37,38]^ It shows that the treatment of adenomyosis with GnRH-a on the basis of dienogest can better reduce the UV of patients. The reduction of UV is meaningful to improve the clinical symptoms of dysmenorrhea and improve the normal implantation rate of fertilized eggs.

Although the overall AE rate did not differ between combination therapy and monotherapy, safety remains a critical issue in long-term management of adenomyosis. Reported AEs were mainly mild to moderate (e.g., irregular bleeding, amenorrhea, vasomotor symptoms, mood changes) and were consistent with the known pharmacology of dienogest and GnRH-a. The lack of increased AE rates suggests that combining the 2 agents does not exacerbate toxicity; however, the small number of studies and limited reporting of AE subtypes restrict definitive conclusions. Larger studies with standardized safety reporting are needed to better define the comparative safety profile.

The incidence of adenomyosis is high. Pelvic endometriosis will secrete inflammatory factors and affect the implantation of fertilized eggs, increasing the risk of infertility. This MA further evaluated the relationship between adenomyosis and pregnancy outcomes. As against non-adenomyosis people, people with adenomyosis had markedly increased abortion, PRM, PTB, and SGA fetuses rates. This is consistent with Pun’s finding that people with adenomyosis have a markedly higher history of previous miscarriage.^[39]^ People with adenomyosis have impaired myometrial function, and the thickness and hardness of myometrium are increased, which will cause the rise of intrauterine pressure and cervical dysfunction to a certain extent, and then cause PRM or natural PTB. Moreover, the increase of UV and uterine cavity pressure will stimulate the secretion of inflammatory substances such as prostaglandins and cause uterine contraction, which may also be the potential factor causing PRM or natural PTB.^[40,41]^ In addition, the rate of SGA fetuses increases in people with adenomyosis, which may be caused by uterine wall trauma, placental insufficiency, abnormal estrogen levels, gestational diabetes mellitus, or gestational hypertension because of factors such as multiple pregnancies and induced abortions. The increase of uterine cavity volume in people with adenomyosis will also affect the normal development of the fetus and cause the occurrence of small fetus. Shakki Katouli et al^[42]^ pointed out that the occurrence of adenomyosis was markedly correlated with cesarean scar defects. After the uterine body is opened, it will cause endometrial trauma to a certain extent, and cause the ectopic endometrium to reach the myometrium, leading to the occurrence of adenomyosis. Therefore, the relationship between adenomyosis and abortion, PTB, CS, etc should be considered in the subsequent clinical research.

The relationship between adenomyosis and adverse pregnancy outcomes may be explained by several pathophysiological mechanisms. Adenomyosis is characterized by invasion of endometrial glands and stroma into the myometrium, accompanied by smooth muscle hyperplasia and chronic inflammation. These alterations can impair endometrial receptivity, disrupt normal myometrial contractility, and increase intrauterine pressure. Such changes contribute to cervical dysfunction, premature rupture of membranes, preterm uterine contractions, and abnormal placentation, thereby increasing the risks of spontaneous abortion, preterm birth, and small-for-gestational-age infants. In addition, uterine wall trauma and remodeling associated with adenomyosis may predispose women to cesarean delivery. Combination therapy with dienogest and GnRH-a could mitigate some of these risks by suppressing estrogen-driven proliferation, reducing lesion activity and uterine volume, improving endometrial receptivity, and decreasing local inflammation. These mechanisms suggest that beyond symptom control, effective medical management of adenomyosis may also have a positive impact on pregnancy outcomes, although further mechanistic and prospective clinical studies are required to confirm this hypothesis.

Beyond statistical significance, these findings carry clear clinical implications. The reduction in dysmenorrhea severity with combination therapy reached a clinically meaningful magnitude, suggesting improved symptom control, reduced analgesic use, and better daily functioning. The modest increase in hemoglobin may alleviate anemia-related fatigue and lessen the need for iron supplementation or transfusion. Decreases in CA-125 levels and uterine volume at longer follow-up indicate suppression of disease activity and structural regression, supporting sustained symptom relief, lower recurrence risk, and potentially improved fertility outcomes. Importantly, these benefits were achieved without an increase in adverse events, underscoring a favorable balance between efficacy and safety. Overall, dienogest combined with GnRH-a may reduce treatment burden and enhance quality of life in women with adenomyosis.

This study has several limitations. First, most of the included studies were observational, single-center, and non-randomized with relatively small sample sizes; only 5 studies with 520 patients contributed to the therapeutic efficacy analysis. The lack of adequately powered randomized controlled trials (RCTs) reduces the strength of causal inference and increases the risk of selection and publication bias. Second, the included studies varied in design (cohort vs controlled trials), treatment regimens (dosage, sequence, and duration of dienogest and GnRH-a), follow-up periods (6, 12, and 18 months), and patient characteristics such as demographics or disease severity. This methodological and clinical heterogeneity may have contributed to the statistical heterogeneity observed in several outcomes and limits the robustness and external validity of our conclusions. Third, the efficacy indicators in this meta-analysis (VAS, Hb, CA-125, and uterine volume) primarily capture pain relief and selected biological or structural changes, but they do not fully reflect overall therapeutic benefits, particularly in terms of patient-reported outcomes and quality of life, which were inconsistently assessed. Finally, although funnel plots did not show major asymmetry, the small number of included studies reduces the ability to reliably detect publication bias.

Taken together, these limitations suggest that the current meta-analysis can provide theoretical guidance but does not yet constitute a universal evaluation applicable to routine clinical practice. The results may be most applicable to women with adenomyosis who wish to preserve fertility, present with moderate-to-severe symptoms, or have larger uterine volumes and elevated CA-125 levels, in whom longer treatment durations may yield more pronounced benefits. In contrast, caution should be exercised in women actively planning pregnancy, in perimenopausal women at risk of hypoestrogenic complications, or in patients with significant comorbidities, where the risk–benefit profile of combination therapy remains uncertain. Future large-scale, multicenter randomized controlled trials with standardized protocols, longer follow-up, and inclusion of patient-reported outcomes are needed to validate these findings and clarify their applicability across broader patient populations.

## 5. Conclusion

This meta-analysis confirmed that dienogest plus GnRH-a can relieve dysmenorrhea, improve hemoglobin levels, and reduce uterine volume in women with adenomyosis. Previous abortion, preterm birth, and cesarean section were also identified as risk factors associated with the disease and adverse pregnancy outcomes. Nevertheless, evidence on the long-term efficacy and safety of combination therapy is limited, as most studies had short follow-up and inconsistent reporting of side effects. Thus, our results provide theoretical guidance but should be interpreted with caution, and future large-scale trials with longer follow-up are needed to validate sustained benefits and clarify long-term risks.

## Author contributions

**Conceptualization:** Yan Wang, Xiye Wang, Leyi Zhang.

**Data curation:** Yan Wang, Leyi Zhang.

**Formal analysis:** Xiye Wang, Leyi Zhang.

**Funding acquisition:** Yan Wang.

**Investigation:** Yan Wang, Xiye Wang, Leyi Zhang.

**Methodology:** Yan Wang, Xiye Wang, Leyi Zhang.

**Supervision:** Yan Wang, Leyi Zhang.

**Validation:** Yan Wang, Xiye Wang, Leyi Zhang.

**Visualization:** Yan Wang, Xiye Wang, Leyi Zhang.

**Writing – original draft:** Yan Wang, Leyi Zhang.

**Writing – review & editing:** Yan Wang, Leyi Zhang.
